# Severely malnourished children with a low weight-for-height have a higher mortality than those with a low mid-upper-arm-circumference: III. Effect of case-load on malnutrition related mortality– policy implications

**DOI:** 10.1186/s12937-018-0382-6

**Published:** 2018-09-15

**Authors:** Emmanuel Grellety, Michael H. Golden

**Affiliations:** 1 Research Center Health Policy and Systems - International Health, School of Public Health, Université Libre de Bruxelles, Bruxelles, Belgium; 2 Department of Medicine and Therapeutics, University of Aberdeen, Aberdeen, Scotland

**Keywords:** Nutrition, Acute malnutrition, Severe acute malnutrition, SAM, Mid-upper-arm circumference, MUAC, Weight-for-height, WHZ, Mortality, Case fatality rate, Wasting, Oedema, Kwashiorkor, Diagnosis, Case load, Prognosis, Child, Human

## Abstract

**Background:**

Severe acute malnutrition (SAM) is diagnosed when the weight-for-height Z-score (WHZ) is <−3Z of the WHO_2006_ standards, or a mid-upper-arm circumference (MUAC) of < 115 mm or there is nutritional oedema. Although there has been a move to eliminate WHZ as a diagnostic criterion we have shown that children with a low WHZ have at least as high a mortality risk as those with a low MUAC. Here we take the estimated case fatality rates and published case-loads to estimate the proportion of total SAM related deaths occurring in children that would be excluded from treatment with a MUAC-only policy.

**Methods:**

The effect of varying case-load and mortality rates on the proportion of all deaths that would occur in admitted children was examined. We used the same calculations to estimate the proportion of all SAM-related deaths that would be excluded with a MUAC-only policy in 48 countries with very different relative case loads for SAM by only MUAC, only WHZ and children with both deficits. The case fatality rates (CFR) are taken from simulations, empirical data and the literature.

**Results:**

The relative number of cases of SAM by MUAC alone, WHZ alone and those with both criteria have a dominant effect on the proportion of all SAM-related deaths that would occur in children excluded from treatment by a MUAC-only program. Many countries, particularly in the Sahel, West Africa and South East Asia would fail to identify the majority of SAM-related deaths if a MUAC only program were to be implemented. Globally, the estimated minimum number of deaths that would occur among children excluded from treatment in our analyses is 300,000 annually.

**Conclusions:**

The number, proportion or attributable fraction of children excluded from treatment with any change of current policy are the correct indicators to guide policy change. CRFs alone should not be used to guide policy in choosing whether or not to drop WHZ as a diagnostic for SAM. All the criteria for diagnosis of malnutrition need to be retained. It is critical that methods are found to identify those children with a low WHZ, but not a low MUAC, in the community so that they will not remain undetected.

**Electronic supplementary material:**

The online version of this article (10.1186/s12937-018-0382-6) contains supplementary material, which is available to authorized users.

## Background

Severe acute malnutrition (SAM) is a lethal condition accounting for about half to one million childhood deaths [1] annually for children with a weight-for-height/length (WHZ) below the recommended WHO cut-off. If children with the other WHO definitions of SAM are added the death toll is much larger. Identification and treatment of all children with any of the current definitions of SAM mandated by the World Health Organisation (WHO) is a public health priority.

The WHO defines SAM using three independent criteria, WHZ of <−3Z of the WHO_2006_ growth standards, an absolute mid-upper-arm circumference (MUAC) of < 115 mm or the presence of nutritional oedema [2, 3]. Some children satisfy several of these criteria.

MUAC can be easily and quickly measured using a simple coloured tape around the upper arm and oedema can also be easily assessed in the field. On the other hand, assessment of WHZ requires the weight and height to be taken and the resulting numbers looked up in tables. There is no doubt that MUAC is much easier to assess than WHZ. For reasons of speed, convenience, cheapness and simplicity MUAC has been used for many years to assess malnutrition [4–8]. The ease of use makes community screening for SAM with MUAC practical and has been a great advance in identifying affected individuals in the community.

However, there has now arisen a concerted movement to stop the assessment of WHZ altogether, even in hospitals and clinics where it is routinely measured at present. The advocates for only using MUAC are adamant that any research to develop innovative methods to assess WHZ in the community is a “*waste of effort*” as MUAC is the only criterion that is needed [9–11]. We examined community based survey data from 48 countries and find that only 16.5% of children who fulfil the WHO definitions of SAM meet both the MUAC and WHZ criteria. If WHZ is abandoned as a criterion about 45% of children with SAM by WHZ alone will fail to be identified because their MUAC is above 115 mm. Which criterion identifies the majority of SAM children varies dramatically from country to country and the two criteria identify different individuals. For these reasons we advocated that both MUAC and WHZ continue to be routinely used to assess children for SAM and, critically, that convenient and simple ways to assess WHZ in the community to identify children with only a deficit in WHZ but not MUAC has to be a major research priority [12].

These suggestions met with a forceful criticism from a multi-authored paper [9] which appears to have widespread support by both agencies and donors [13]. The putative basis of the opinion that WHZ should not be used at all was that anything that diverts resources from the widespread use of MUAC to identify SAM would hinder its implementation and therefore WHZ assessment must be suppressed [9, 14]. The reasons given against the use of WHZ did not simply emphasise its inconvenience, with which we agree. The following were asserted: 1) children with a low WHZ are healthy; 2) their low WHZ is due entirely to their having longer legs so they do not require treatment; 3) WHZ is a poor predictor of mortality in children; 4) MUAC is a good predictor of mortality in children; 5) the two diagnostic parameters are not complementary; and 6) addition of WHZ does not improve the sensitivity or specificity of future all-cause mortality prediction with MUAC. These contentions were robustly refuted [15].

We have shown in the two preceding papers [16, 17], 1) that WHZ < −3Z carries as high, or higher, risk of death as MUAC < 115 mm; they are clearly not “healthy” and undeserving of treatment. 2) That the two parameters not only identify different children, and therefore different risks, but also children satisfying both criteria have a higher mortality showing the defects to be additive. 3) That “long legs” is an inadequate explanation for the regional difference in SAM by WHZ [12, 18, 19]. 4) That all the data previously analysed by comparison of ROC curves, and relied upon to make the assertions of MUAC’s superiority are severely biased because of mathematical coupling [20, 21] as well as stochastic and other problems of interpretation [15]. 5) Despite the flaws the data actually show that WHZ carries a higher mortality risk than MUAC when appropriately analysed [16]. Indeed, there are abundant data to confirm that WHZ < −3Z carries a substantial risk of death [22–26], but these papers did not measure MUAC for comparison. Thus, all the criticisms asserted by Briend et al., and repeated [9, 14, 27, 28] are, in our opinion, incorrect. Nevertheless, their advocacy has led most humanitarian agencies and some Governments to abandon WHZ altogether.

We do agree that WHZ is more inconvenient and difficult to measure than MUAC; but this is the *only* legitimate criticism of widespread use of WHZ. The question arises as to the potential fate of the ≈45% of children who would not be identified if WHZ measurement was omitted completely.

Having shown that the case fatality rates (CFRs) are not lower in children with only a deficit in WHZ, this paper examines the practical programmatic differences between a MUAC-only program and a complete program.

The object of this study was to estimate the proportion and where possible the numbers of all SAM related deaths that would occur in children who would be excluded from treatment if a MUAC-only program replaced a complete program.

## Methods

### Effect of case-load

We used a simple excel spreadsheet to demonstrate the effect of variations of the proportions of the total case-load comprised of children with SAM by MUAC, WHZ and by both MUAC and WHZ with their corresponding CFRs on the proportion of SAM deaths that would occur in excluded children if a MUAC-only program was used.

The total SAM-related-deaths is given by:

(**M**_*CL*_**x M**_*CFR*_ + **W**_*CL*_**x W**_*CFR*_ + **B**_*CL*_**x  B**_*CFR*_)

Where M = children with MUAC < 115 mm and WHZ > −3Z (S-muac): W = children with WHZ < −3Z and MUAC > 115 mm (S-whz): B = children with “Both” MUAC < 115 mm and WHZ < −3Z (S-both): subscript *CL* = the proportions of the total case load of SAM that are in categories M, W and B: subscript *CFR* = Case fatality rates for children with M, W and B. The case load always sums to 100% of SAM children (i.e. SAM due to oedema, kwashiorkor with or without wasting, is not considered in this calculation).

Then the proportion of total SAM-related-deaths that would occur in children that would **not** be eligible for admission and treatment if WHZ were to be dropped as an admission criterion is given by:

**1** − (**M**_*CL*_**x M**_*CFR*_ + **B**_*CL*_**x B**_*CFR*_)/(**M**_*CL*_**x M**_*CFR*_ + **W**_*CL*_**x W**_*CFR*_ + **B**_*CL*_**x B**_*CFR*_).

For the simulation, the relative case-loads were varied from zero children with S-muac to zero children with S-whz. The remainder of the children either had the alternative criterion or had S-Both. The proportion of children with S-both was varied from 10 to 30% (the limits we found in representative nutritional surveys [12]). The CFRs for S-muac, S-whz and S-both were examined by changing the ratio of S-muac to S-whz mortality from half to twice the mortality of the other to represent the likely limits of the variation in mortality risk. S-both’s CFR was set at the sum of the CFRs of S-muac and S-whz in accordance with the empirical data and most of the literature reports [16, 17]. Variation of the overall CFR will affect the total number of deaths, but the proportions of SAM-related deaths which would be eligible or ineligible for treatment is not affected by the absolute CFRs, only by their ratios and relative case-loads. Thus, if the sizes of the three CFRs and the proportions of the three case loads do not change the percent of children that will become ineligible for treatment does not change when the total SAM-related death rate rises or falls.

### Proportion of all SAM-related-deaths that would occur in children ineligible for treatment with a MUAC-only program by country

The literature and patient data reported in the first and second papers [16, 17] were subject to ascertainment bias which made the proportions of the case load coming from the different categories unrepresentative of SAM in the community. In particular, the proportion of children in the S-both category was much higher than that found in the community. That is, the case load ratios of S-muac: S-whz: S-both differed significantly from that found in representative community surveys of malnourished children [9]. For that reason the case load ratios of S-muac, S-whz and S-both reported in papers I and II [16, 17] were not used in any calculation. To fairly represent the situation of SAM children in the community we used the data previously published from representative community surveys [12]. These ratios are derived from analysis of 48,697 SAM children out of a total surveyed population of 1,384,068 children, 6–59 months, (1832 surveys) from 48 countries.

The community-derived, proportionate case-load estimates were then used to estimate the proportion of the total deaths that would occur in SAM children with a MUAC-only program; the residue of S-whz would be excluded. As the mortality rates for S-muac, S-whz and S-both that would occur in untreated SAM-children in the communities are unknown we used mortality rates from 3 sources. First, those used in our theoretical simulation; second, those found in paper 1 [16]; third, the relative risks of death derived from the meta-analysis of the literature values where WHO criteria were used and oedematous cases excluded [17]. The forest plots from the meta-analyses comparing S-muac with S-whz and S-both, using adjustment for study quality, [17] are given in Additional file 1. The relative risks of death from S-muac, S-whz and S-both were 1.00: 1.14: 2.70 respectively.

The calculations were the same as for the theoretical simulation.

### How many are affected?

In order to estimate the number of children excluded by a MUAC-only program we examined data from a global estimate of SAM-related deaths [1] and from India [29]. These estimates are minimum estimates because they were based upon prevalence data rather than incidence data and only on WHZ data. We used case loads of S-muac, 39.5%, S-whz 44.0% and S-both 16.5% for the global SAM-deaths estimate and S-muac 15.5%, S-whz 61.6% and S-both 22.9% for India [12]. The CFRs were the same for the single deficits and double for S-both.

### Ethical statement

This analysis used published data only thus no formal ethical clearance was required.

## Results

### Theoretical considerations

**Fig. 1:**
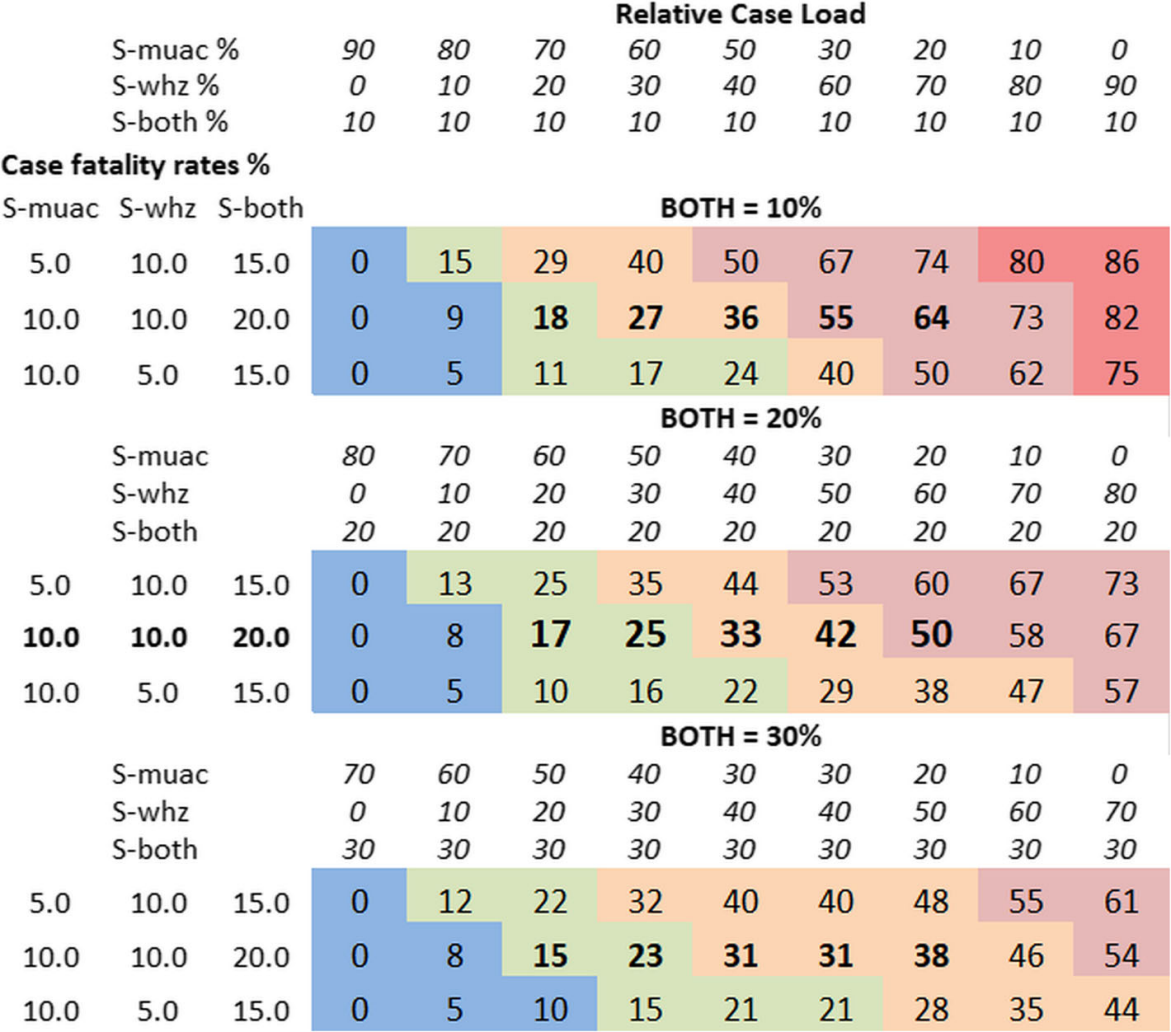
Percentage of SAM-related-deaths of children that would be excluded from treatment in a MUAC-only program. Simulation of the effect of various case loads and case fatality rates for children with SAM by MUAC-only, WHZ-only and Both criteria. The data gives the percent of total SAM-related-deaths that would occur in children excluded from treatment in a MUAC-only program. Case Loads and Case Fatality Rates for: S-muac = MUAC < 115 mm with WHZ > = −3Z: S-whz = WHZ < −3Z with MUAC > 115 mm: S-both = MUAC < 115 mm and WHZ < −3Z. The colours represent the percent of deaths occurring in cases that would be excluded from treatment in such a program: Red 75–100%: Pink 50–75%: Orange 25–50%: green 10–25%: Blue 0–10%

Figure 1 illustrates the effect of variation of the case-loads of S-muac, S-whz and S-both and corresponding CFRs to derive the percent of deaths occurring in children excluded by a MUAC-only program. The three lines in each block show the effect of S-muac CFR being half, the same or twice the CFR of S-whz. These CFRs represent the likely relative risk and the outside limits. The three blocks show the effect of an increasing S-both percentages.

If a WHZ-only program was used the results would be the exact inverse of the percentage exclusion shown.

With a MUAC-only policy, if there are no children in the community with S-whz (col 1) then all the SAM children will be identified. If there are no children with S-muac (last col) the only deaths of MUAC children will be those who also have S-whz, i.e. those with S-both. There will be slightly more deaths than the proportion of overlap because of the higher mortality risk of S-both children.

A likely scenario is given in block two, second row. As the percentage of S-muac in the community decreases from 60 to 20% the deaths that occur in excluded children increases from 17 to 50%. To have 20% S-muac is frequently found in nutritional surveys from some regions [12]. Figure 1 also shows that the exclusion rate is reduced with more S-both children. For example, if 50% of the children have S-muac (column 5) as S-both increases from 10 to 20 to 30% the relative proportion of deaths of excluded children decreases from 36, to 33 to 31% respectively. As the proportion of S-muac decreases the effect of S-both on excluded cases increases; thus, were there is 20% of S-muac children (col 7) the percent of excluded children falls from 64 to 50% to 38%.

In contrast, a change of CFR ratios from half to twice has a relatively minor effect on the proportion of excluded children (compare the 3 rows vertically). Thus, a change of case-load ratio is more important than a change in CRFs ratio within the ranges reported [16, 17] in determining the extent of exclusion of S-whz children.

It should be emphasised that these simulations compares the deaths in excluded children (S-whz) with all the children that would be identified using a MUAC measurement (i.e. S-muac plus S-both). In papers [16, 17], CFRs from S-muac and S-whz were compared. Here, by combining S-both with S-muac in the calculations we replicate the actual effect of only measuring MUAC on the proportion of deaths related to SAM that would be excluded from treatment or considered in a coverage survey.

### Country data

**Fig. 2:**
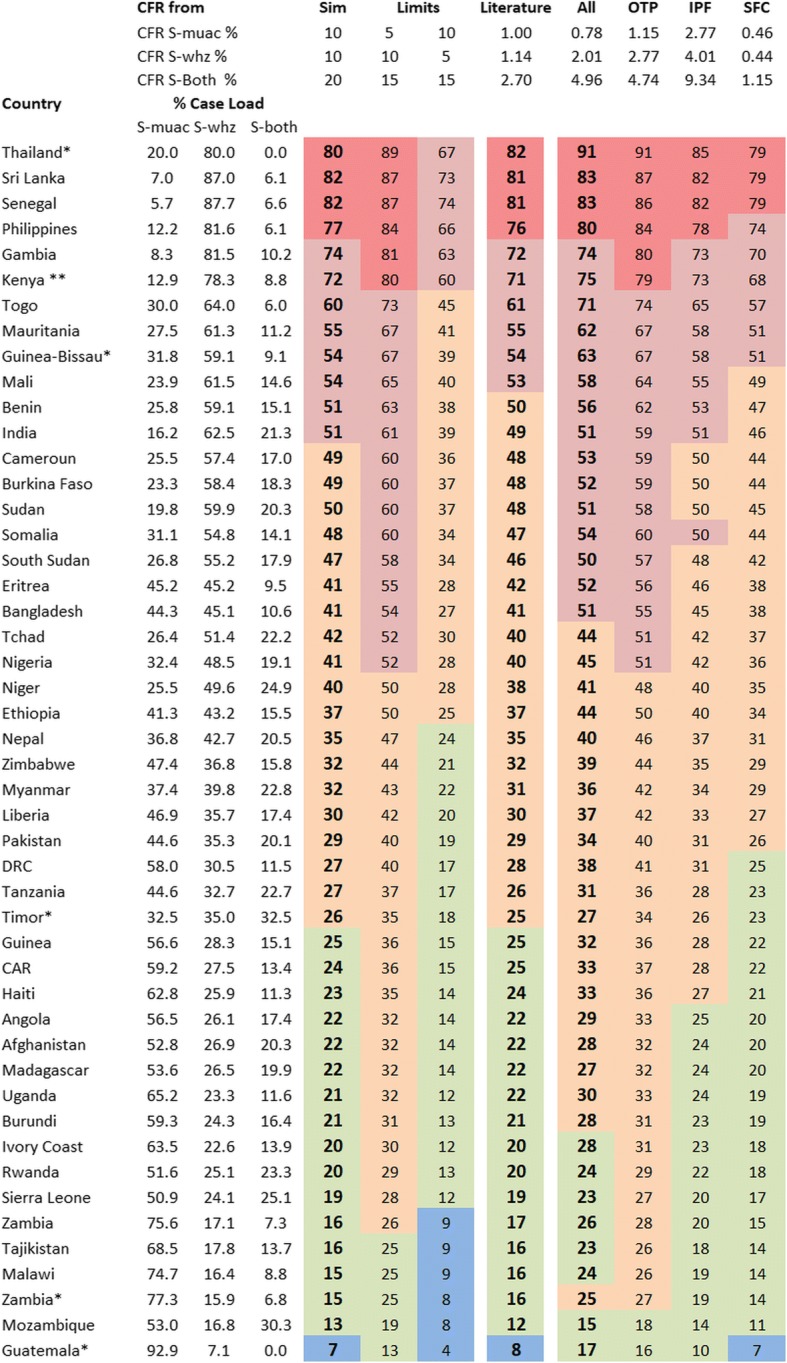
Percentage of SAM-related-deaths of children that would be excluded for treatment with a MUAC-only program by country. *Sim* simulation data from Fig. 1, representing the probable ratio of case fatality rates (CFRs) and likely extremes; *All, IPF, OPT, SFC* are the empirical case fatality rates of patients under different modes of treatment [16]; *Literature* mortality rates from Additional file 1 derived from the data in reference [17]; *Case Loads* S-muac = MUAC < 115 mm with WHZ > = −3Z: S-whz = WHZ < −3Z with MUAC > 115 mm: S-both = MUAC < 115 mm and WHZ < −3Z; *DRC* Democratic Republic of the Congo; *CAR* Central African Republic. The case loads per country are from reference [12]. The colours represent the percent of total SAM-related-deaths occurring in cases that would be excluded from treatment in a MUAC-only program: Red 75–100%: Pink 50–75%: Orange 25–50%: green 10–25%: Blue 0–10%. * These countries case load comes from a small sample size. ** The case load from Kenya comes from the North of Kenya (similar to Sahel)

Because the mortality ratios in nearly all communities is unknown, in Fig. 2 we have used the CFRs from the simulation and estimated from papers I and II [16, 17]. These CFRs are then combined with the actual country case-loads found in community nutrition surveys published previously [12].

In Fig. 2 we present, by country, the estimates of the percentage of deaths of SAM children that would occur in children excluded from treatment if only MUAC measurements are taken. There is reasonable agreement between the estimates based upon the different CFRs. For example, in Senegal the three main CFR estimates indicate that 82, 81 and 83% of deaths occur in excluded children; whereas, in Mozambique only 13, 12 and 15% of deaths occur in excluded children. Taking the average of the empirical and literature exclusion rates, 12 countries would exclude more than three quarters and 34 would exclude more than half of the SAM children that die. Only 3 of the 48 countries would include more than 80% of children who die with SAM.

The corresponding analysis using a WHZ-only program is given in Additional file 2. It is clear that a WHZ-only program would also fail to identify a large proportion of the children at high risk of death.

**Fig. 3:**
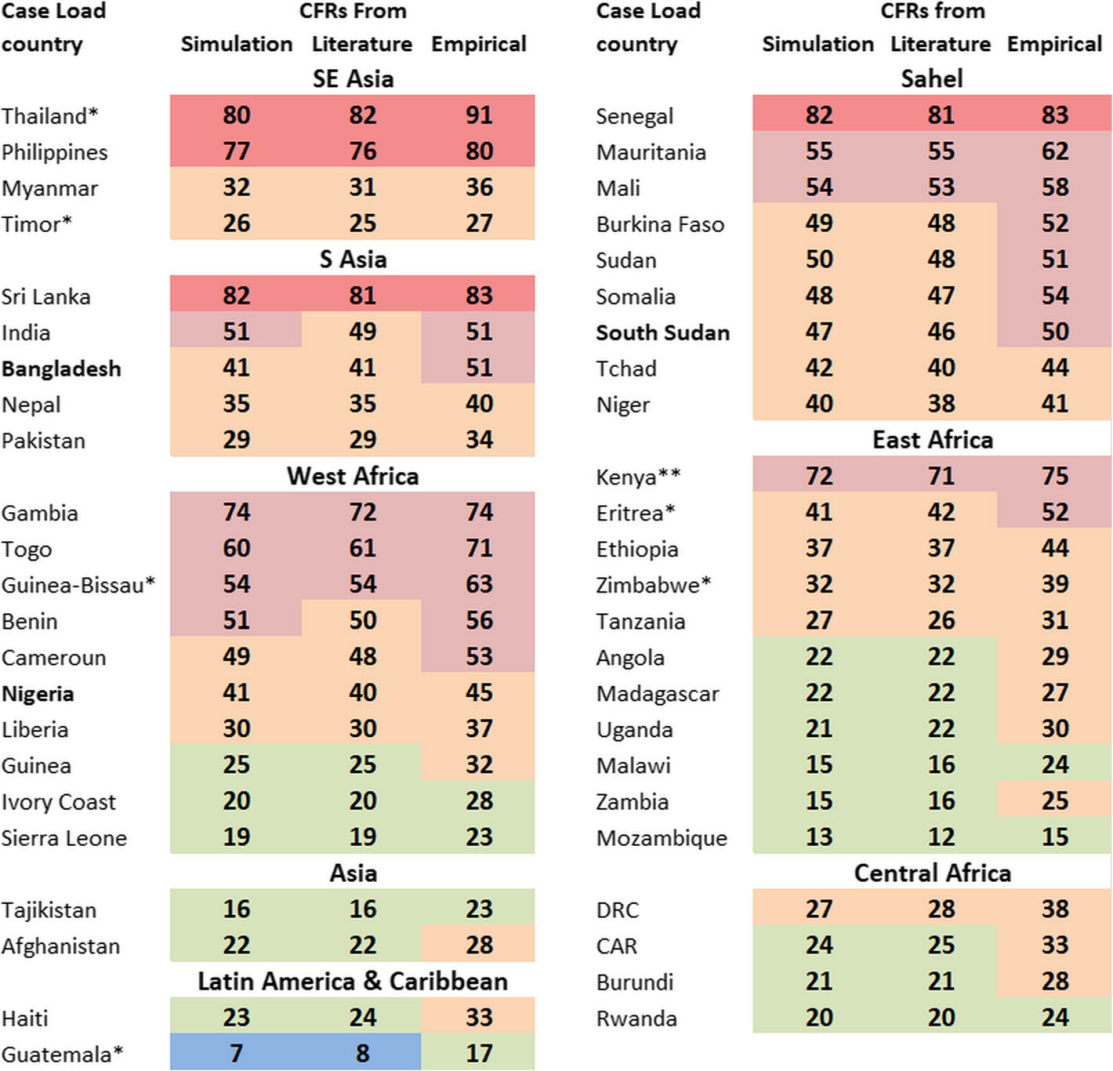
Percentage of SAM-related-deaths of children that would be excluded for treatment with a MUAC-only program by Region. *Simulation* see Fig. 1; *Literature* data from [17]; *Empirical* data from [16]; *Case load* data from [12]; *CFRs* Case fatality rates; *SE Asia* South East Asia; *S Asia* South Asia; *DRC* Democratic Republic of the Congo; *CAR* Central African Republic. The colour code is the same as Figs. 1 and 2. * Case load ratios from these countries is based on a small sample size. ** For Kenya the case load data comes from the North of Kenya (could be counted with Sahel). In bold are some of the countries whose governments have officially adopted a MUAC-only program (2017)

The countries are grouped by region in Fig. 3. If the countries of South & South East Asia and the Sahel were to adopt a MUAC-only policy then a substantial proportion of SAM children’s deaths would occur in excluded children. The same applies in many of the countries in West Africa. Some of these countries are characterised by a dry Sahalian type interior and a wet, heavily populated coast. These ecologically different areas may have different levels of exclusion of S-whz so that the effect of only measuring MUAC may be more deleterious in some areas than others, and may give a within-country bias to nutritional surveys aimed at establishing the prevalence of SAM and the level of exclusion. The same conditions apply to some of the East African countries. On the other hand several countries in West, East and Central Africa as well as Asia and Latin America would exclude less than 25% of children that contribute to SAM mortality.

### Numbers of excluded SAM children who die

Conversion of relative case loads and CFRs into the number of deaths of SAM children who will be ineligible for any treatment under a MUAC-only program is shown in Table 1. Using Black et al.’s [1] estimate of global SAM deaths of 540,000 calculated from WHZ prevalence, the total deaths increases to over 800,000 when we include S-muac deaths. Of these over 300,000 children (38%) will die without the possibility of treatment if WHZ is not measured. In India, although Black et al. estimated that there would be 145,000 deaths, Mohan & Mohan [29] estimated the actual number of deaths due to SAM to be 270.000; of these, more than half, 155,000, would be excluded with a MUAC-only policy.

**Table 1:** Estimation of the possible number of deaths from SAM that would be missed using a MUAC only program

	WHZ deaths	Total deaths	WHZ only (S-whz)	MUAC only (S-muac)	Both criteria (S-both)	MUAC-only %missed	WHZ-only %missed
Global	540,000^a^	817,000	309,000	277,000	231,000	37.8	33.9
India	270, 000^b^	309,000	155,000	39,000	115,000	50.2	12.6

The estimates of total deaths and proportions were derived as in methods. *WHZ deaths* deaths estimated by WHZ < −3Z (i.e. S-whz + S-both); *Total deaths* WHZ deaths plus MUAC-only deaths based on ratios found in reference [12] for Global and India (S-both = 16.5 and 22.9%). Equal mortality risk for S-whz < −3Z and S-muac < 115 mm and twice the mortality risk for S-both is assumed. ^a^ Data from reference [1]; ^b^ Data from reference [29]

## Discussion

If the primary objective of treating children with SAM is to prevent death then it is logical to look at the percent of deaths that occur in SAM children that would be excluded from treatment with any change in policy. This should then determine whether or not a policy change is unacceptable. This information cannot be obtained from comparison of CFRs by regression or areas under ROC curves.

The CFRs estimated from our empirical data [16] and a meta-analysis of the literature [17] are consistent and show that the CFRs for S-muac and S-whz are not sufficiently different to affect the rate of exclusion when only MUAC is used for SAM diagnosis. The dominant factor is the case-load mix because even when the CFRs differ substantially the numbers of children that are excluded show relatively minor changes.

The present country data, by themselves, cannot be used to determine the absolute numbers of children calculated to die or that would be excluded because of a change in policy. This requires our data to be combined with the prevalence/incidence rates, population size and community mortality rate for at least one of the diagnostic groups. To then derive population attributable fractions also needs the relative risks of death from SAM children using the non-malnourished children in the same community as the reference [30, 31]. The results comparing Black et al. [1] and Mohan & Mohan [29] demonstrate the difficulties in arriving at accurate estimates. Nevertheless, the numbers of children who die with SAM who would be ignored, if only MUAC is used is massive. We estimate this to be about 40% of all SAM children’s deaths globally; but this is variable by country and region as Fig. 3 shows. Ignoring these children and their deaths is the real cost of promoting a MUAC-only program, and begs the question of “what is acceptable” from a humanitarian point of view. Reliable and appropriate estimates are essential if correct priorities and policies are to be set by Governments to address SAM. Death is not the only adverse effect of severe malnutrition. There are other major health and long term consequences of failing to identify and treat the very much larger number of children with SAM that do not die, estimated to be 10 million in India by WHZ [29].

One answer to the problem of excluded children could be to increase the MUAC cut-off point. This is a simplistic suggestion that is impractical as it would then include a very large proportion of the whole childhood population at a much lower risk of death [9, 13, 28, 32, 33] and divert and dilute the attention needed for the high risk children.

The paper from Uttar Pradesh, India, by Kapil et al. [34] is germane to addressing this suggestion. SAM in Uttar Pradesh by WHZ was 2.2%; when MUAC was added the prevalence increased to 2.5%. If the MUAC cut-off was increased to 135 mm then 17% of all the children in the population would need to be identified; however, 12% of the S-whz children would still be missed and the extra case load would only identify a further 15% of S-whz. Five in 6 of the extra children would be “false positives” for SAM. Our unpublished analysis from Africa is in agreement with these figures. The cost, logistics, staff time with inevitable disruption to other essential medical services, add-on costs for the parents, possible family guilt or stigma concerning the need to be checked for SAM and risk of bringing the program into disrepute all mitigate against this policy. Elsewhere evidence shows that there are several major difficulties when a false positive rate exceeds the true positive rate [35, 36]. Furthermore, WHO now recommends “*not to provide formulated supplementary foods on a routine basis to children who are moderately wasted*” [37].

In view of the evidence, why has MUAC morphed from a simple and effective community screening program into a MUAC-only program? Is it really necessary for total suppression of WHZ as a diagnostic for SAM to legitimise the use of MUAC?

We suggest that there are several reasons. First, based upon the data presented in the first two papers of this series [16, 17] as well as the present paper it is clear that an inappropriate statistical strategy has been exclusively used in the past using ROC curve analysis of entire populations to compare the relative CFRs [15]; when the risks are suffered by different children entirely, the risks are also different, making comparisons for diagnostic purposes largely meaningless. Second, the exclusive focus on CFRs alone and the notion that MUAC is a “superior” test; it can only be superior if it identifies the same risk. Third, the neglect of case load in determining the numbers of excluded children or the calculation and use of further derived statistics such as population attributable fraction. Fourth, repeated assertions that it is safe to ignore S-whz children because they are healthy when there are no data to support this contention and abundant data to show that these children are at high risk of death combined with misquotation and criticism of any data that does not support the proposition [38]. Fifth, by forceful advocacy to donors, research funding agencies, UN agencies and many in the humanitarian organisations [13]. Sixth, because of an understandable desire to make everything as simple as possible whilst denying there is any cost of excluding SAM children [26]. Simplification beyond what is possible renders programs unworkable or unethical (try removing the cold chain from vaccination services). An effort to simplify SAM treatment by suppressing use of F75, the initial diet designed for the most critically ill of children [39] was dropped when the reasons for F75 were properly explained to the agencies. Only using MUAC is certainly simple, but it has a real cost, and that is measured in lives lost. Last, because of cognitive biases, particularly confirmation bias [40] among those subscribing to a MUAC-only policy and those providing “confirmatory” low quality evidence such as some of the papers reviewed in [17].

The ease of use of MUAC makes community screening to identify children in need to SAM treatment practical and has led to increasingly greater “coverage” rates for those children with MUAC < 115 mm. Our data does not question the utility of this, what is does demand is research to find ways that are sufficiently simple to be applied in the community so that children with WHZ < − 3 can also be included in treatment programs. Stereo-photography has been used for many years [41] but with modern technology this has become practical [42–44]. There are reports of low cost scanning attachments to smart-phones that give precise measures of height, head-circumference and MUAC [45]. Therefore, on the horizon are techniques that will make the assessment of WHZ simple to use in the community. Such studies must be properly funded, supported and then implemented and make it premature to cease considering WHZ as a proper diagnostic for SAM.

## Conclusions

Some within the nutritional community has been misled by replicated but flawed analyses and assertions. They are also attracted by the ease and low cost of MUAC screening; these practical aspects are clearly advantages to be considered. MUAC-only programs fail to identify enormous numbers of the most vulnerable children in many societies. It may be difficult to identify S-whz children, but that is not a reason to pretend these children do not exist or to justify ignoring them by making false claims such as that they are healthy. It is essential that they are included in any program that claims to address the scourge of SAM. In our opinion many of these programs should be considered as contravening the dictates of Hippocrates.

Both a WHZ < −3Z and MUAC < 115 mm must be retained and used wherever possible as diagnostic criteria for SAM. The research priority must be to develop innovative ways of assessing WHZ so that it can be extended to S-whz identification in the community.

## Additional files

Additional file 1: **Figure S1.** Forest plots of papers 1–7 of [17] to determine the CRFs of children with S-muac, S-whz and S-both. The meta-analyses were performed as in [17] using the quality of the study as weighting (QE); only reports that used the recommended WHO diagnostic criteria, and excluded oedematous (oed) cases were selected for this analysis. *IND* India; *NER* Niger; *SDN* South Sudan; *UGA* Uganda; *MWI* Malawi; *SEN* Senegal; *RR* relative risk; CI confidence intervals. (TIF 1732 kb)

Additional file 2: **Figure S2.** Percentage of SAM-related-deaths of children that would be excluded from treatment by a WHZ-only program. *Sim* simulation data from Fig. 1, representing the likely extremes and probable ratio of case fatality rates (CFRs); *All, IPF, OPT, SFC* are the empirical case fatality rates of patients under different modes of treatment [16]; *Literature* mortality rates from Additional file S1, from reference [17]; *Case Loads* S-muac = MUAC < 115 mm with WHZ > = −3Z: S-whz = WHZ < −3Z with MUAC > 115 mm: S-both = MUAC < 115 mm and WHZ < −3Z; *DRC* Democratic Republic of the Congo; *CAR* Central African Republic. The case loads per country are from reference [12]. The colours represent the percent of total SAM-related-deaths occurring in cases that would be excluded from treatment in a MUAC-only program: Red 75–100%: Pink 50–75%: Orange 25–50%: green 10–25%: Blue 0–10%. * These countries case load comes from a small sample size. ** The case load from Kenya comes from the North of Kenya (similar to Sahel). (TIF 4541 kb)

## Abbreviations

CFR: Case fatality rate
IPFs: In-patient treatment facilities
MAM: Moderate acute malnutrition
MUAC: Mid-upper-arm-circumference
NGOs: Non-governmental organisations
OTPs: Out-patient treatment programs
ROC curve: Receiver operating characteristic curve
SAM: Severe acute malnutrition
SFCs: Supplementary feeding centres
WHO: the World Health Organisation
WHZ: Weight-for-height Z-score

## References

[1] Black RE, Victora CG, Walker SP, Bhutta ZA, Christian P, De Onis M (2013). Maternal and child undernutrition and overweight in low-income and middle-income countries. Lancet.

[2] WHO, Unicef. WHO child growth standards and the identification of severe acute malnutrition in infants and children: a joint statement by the World Health Organization and the United Nations Children’s fund. 2009. http://www.who.int/nutrition/publications/severemalnutrition/9789241598163_eng.pdf 24809116

[3] WHO. Guideline: Updates on the management of severe acute malnutrition in infants and children. Geneva, World Health Organization; 2013. http://www.who.int/nutrition/publications/guidelines/updates_management_SAM_infantandchildren/en/. 24649519

[4] Jelliffe DB (1966). The assessment of nutritional status of the community.

[5] Jelliffe EFP, Jelliffe DB (1969). The arm circumference as a public health index of protein-calorie malnutrition of early childhood. J Trop Pediatr.

[6] Jelliffe DB (1970). Arm circumference in children. Lancet.

[7] Shakir A, Morley D (1974). Measuring malnutrition. Lancet.

[8] Shakir A (1975). Arm circumference in the surveillance of protein-calorie malnutrition in Baghdad. Am J Clin Nutr.

[9] Briend A, Alvarez JL, Avril N, Bahwere P, Bailey J, Berkley JA (2016). Low mid-upper arm circumference identifies children with a high risk of death who should be the priority target for treatment. BMC Nutr.

[10] EN-Net. WFH versus MUAC. 2015. Emergency Nutrition Network. http://www.en-net.org/question/1915.aspx

[11] EN-Net. Only MUAC for admission and discharge? 2015. Emergency Nutrition Network. http://www.en-net.org/question/1922.aspx

[12] Grellety E, Golden MH. Weight-for-height and mid-upper-arm circumference should be used independently to diagnose acute malnutrition: policy implications. BMC Nutr 2016, 2: 10. https://bmcnutr.biomedcentral.com/articles/10.1186/s40795-016-0049-7

[13] Bailey J, Chase R, Kerac M, Briend A, Manary M, Opondo C et al. Combined protocol for SAM/MAM treatment. The ComPAS study. Field exchange 2016, 53: 44. http://www.ennonline.net/fex/53/thecompasstudy

[14] Hammond W, Badawi AE, Deconinck H (2016). Detecting severe acute malnutrition in children under five at scale. The Challenges of Anthropometry to Reach the Missed Millions. Ann Nutr Disord Ther.

[15] Grellety E, Golden MH (2016). Response to Briend et al “low mid-upper-arm-circumference identifies children with a high risk of death and should be the priority target for treatment”. BMC Nutr.

[16] Grellety E, Golden MH. Severely malnourished children with a low weight-for-height have a higher mortality than those with a low mid-upper-arm-circumference: I. Empirical data demonstrates Simpson’s paradox. Nutr J. 2018. 10.1186/s12937-018-0384-4 10.1186/s12937-018-0384-4 PMC6138885 30217205

[17] Grellety E, Golden MH. Severely malnourished children with a low weight-for-height have a higher mortality than those with a low mid-upper-arm-circumference: II. Systematic literature review and meta-analysis. Nutr J. 2018. 10.1186/s12937-018-0383-5 10.1186/s12937-018-0383-5 PMC6138903 30217196

[18] Post CL, Victora CG. The low prevalence of weight-for-height deficits in Brazilian children is related to body proportions. J Nutr 2001;131:1290–1296. http://jn.nutrition.org/content/131/4/1290.full 10.1093/jn/131.4.1290 11285340

[19] Roberfroid D, Huybregts L, Lachat C, Vrijens F, Kolsteren P, Guesdon B (2015). Inconsistent diagnosis of acute malnutrition by weight-for-height and mid-upper arm circumference: contributors in 16 cross-sectional surveys from South Sudan, the Philippines, Chad, and Bangladesh. Nutr J.

[20] Archie JP (1981). Mathematic coupling of data: a common source of error. Ann Surg.

[21] Tu YK, Maddick IH, Griffiths GS, Gilthorpe MS (2004). Mathematical coupling can undermine the statistical assessment of clinical research: illustration from the treatment of guided tissue regeneration. J Dent.

[22] Puffer RR, Serrano CV (1973). Patterns of mortality in childhood: report of the inter-American investigation of mortality in childhood, Paho scientific publication no. 262, 1–470.

[23] Puffer RR, Serano CV (1973). The role of nutritional deficiency in mortality: findings of the inter-American investigation of mortality in childhood. Bol Ofic Sanit Panam.

[24] Pelletier DL (1994). The relationship between child anthropometry and mortality in developing countries: implications for policy, programs and future research. J Nutr.

[25] O’Neill SM, Fitzgerald A, Briend A, Van Den Broeck J (2012). Child mortality as predicted by nutritional status and recent weight velocity in children under two in rural Africa. J Nutr.

[26] Olofin I, McDonald CM, Ezzati M, Flaxman S, Black RE, Fawzi WW (2013). Associations of suboptimal growth with all-cause and cause-specific mortality in children under five years: a pooled analysis of ten prospective studies. PLoS One.

[27] Briend, A. Use of MUAC for severe acute malnutrition. CMAM forum 2012. http://citeseerx.ist.psu.edu/viewdoc/download?doi=10.1.1.662.303&rep=rep1&type=pdf

[28] Briend A, Maire B, Fontaine O, Garenne M (2012). Mid-upper arm circumference and weight-for-height to identify high-risk malnourished under-five children. Matern Child Nutr.

[29] Mohan P, Mohan SB (2017). Management of Children with severe acute malnutrition in India: we know enough to act, and we should act now. Indian Pediatr.

[30] Rockhill B, Newman B, Weinberg C (1998). The use and misuse of population attributable fractions. Am J Public Health.

[31] Hanley JA (2001). A heuristic approach to the formulas for population attributable fraction. J Epidemiol Community Health.

[32] Shepherd S, Becquet R. Integrated Treatment Protocol for Acute Malnutrition: A Non Inferiority Trial in Burkina Faso (MUAC-Only). ClinicalTrials.gov 2017. https://clinicaltrials.gov/ct2/show/NCT03027505

[33] Maust A, Koroma AS, Abla C, Molokwu N, Ryan KN, Singh L (2015). Severe and moderate acute malnutrition can be successfully managed with an integrated protocol in Sierra Leone. J Nutr.

[34] Kapil U, Pandey RM, Bansal R, Pant B, Varshney AM, Yadav CP (2018). Mid-upper arm circumference in detection of weight-for-height Z-score below− 3 in children aged 6–59 months. Public Health Nutr.

[35] Wald NJ, Hackshaw AK, Frost CD (1999). When can a risk factor be used as a worthwhile screening test?. BMJ: Br Med J.

[36] Rosenblatt RA (1989). The perinatal paradox: doing more and accomplishing less. Health Aff.

[37] World Health Organization. Guideline: assessing and managing children at primary health-care facilities to prevent overweight and obesity in the context of the double burden of malnutrition. Updates for the Integrated Management of Childhood Illness (IMCI) Geneva (Switzerland): World Health Organization; 2017. http://apps.who.int/iris/bitstream/10665/259133/1/9789241550123-eng.pdf 29578661

[38] Murray C (2005). How to accuse the other guy of lying with statistics. Stat Sci.

[39] Briend A (2001). Management of severe malnutrition: efficacious or effective?. J Pediatr Gastroenterol Nutr.

[40] Kahneman D. Thinking, Fast and Slow. Macmillan, 2011. http://www.math.chalmers.se/~ulfp/Review/fastslow.pdf

[41] Piebson WR (1961). Monophotogrammetric determination of body volume. Ergonomics.

[42] Wells JCK, Ruto A, Treleaven P (2008). Whole-body three-dimensional photonic scanning: a new technique for obesity research and clinical practice. Int J Obes.

[43] Yu W. Development of a three-dimensional anthropometry system for human body composition assessment. The University of Texas at Austin; 2008. https://repositories.lib.utexas.edu/bitstream/handle/2152/17838/yuw.pdf%3Bjsessionid%3D61F89DE75AA927339A6A0909C615A7FE?sequence%3D2

[44] Mikat RP (2000). Chest, waist, and hip circumference estimations from stereo photographic digital topography. J Sports Med Phys Fitness.

[45] Conkle J, Ramakrishnan U, Flores-Ayala R, Suchdev PS, Martorell R (2017). Improving the quality of child anthropometry: manual anthropometry in the body imaging for nutritional assessment study (BINA). PLoS One.

