# Supplementary figures and images for: Severely malnourished children with a low weight-for-height have a higher mortality than those with a low mid-upper-arm-circumference: III. Effect of case-load on malnutrition related mortality– policy implications
Source: Nutr J. 2018 Sep 15;17:81. doi: 10.1186/s12937-018-0382-6 (PMC6138898; doi:10.1186/s12937-018-0382-6)

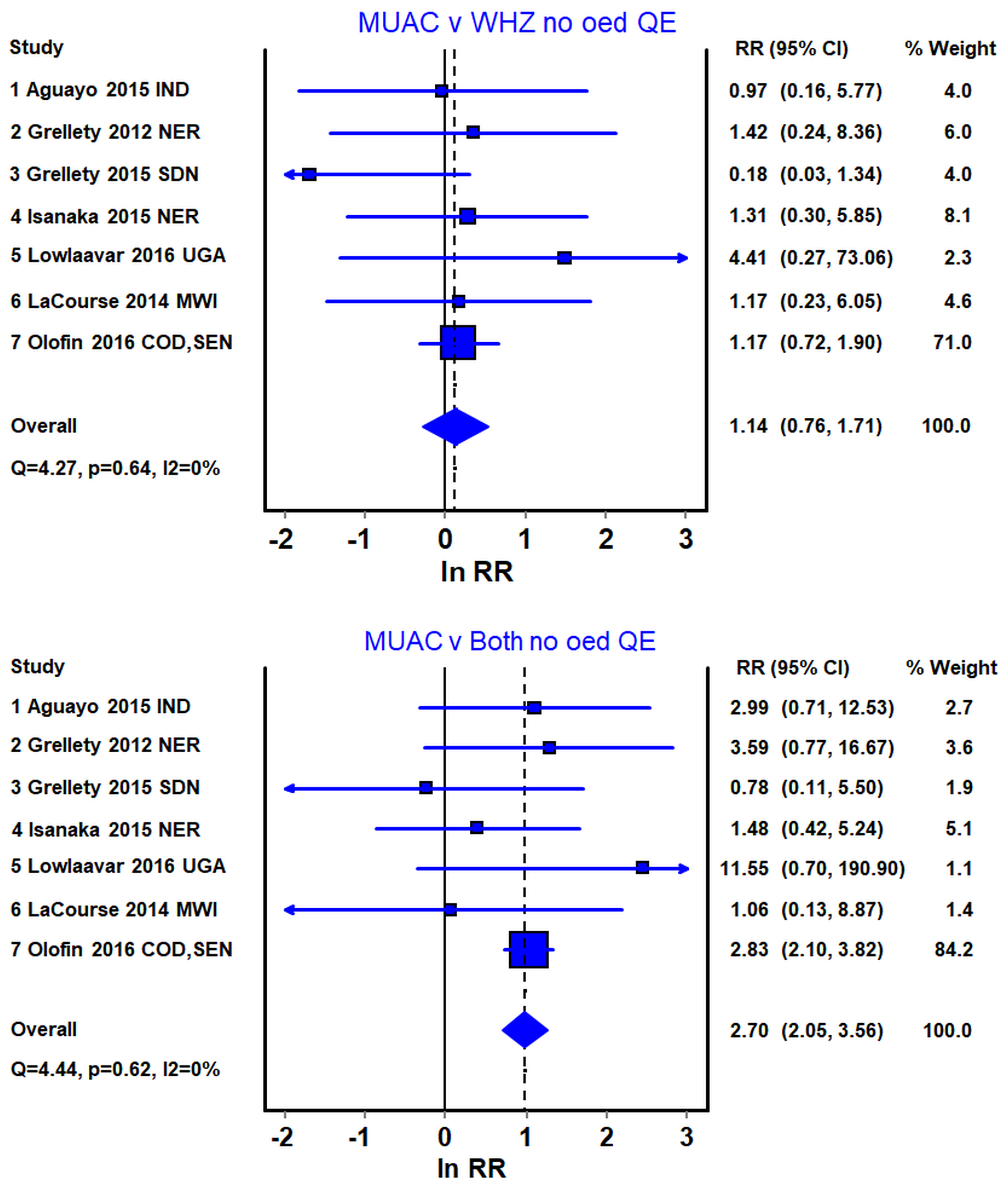

Supplement: Supplementary file 1 — Figure S1. Forest plots of papers 1–7 of [17] to determine the CRFs of children with S-muac, S-whz and S-both. The meta-analyses were performed as in [17] using the quality of the study as weighting (QE); only reports that used the recommended WHO diagnostic criteria, and excluded oedematous (oed) cases were selected for this analysis. IND India; NER Niger; SDN South Sudan; UGA Uganda; MWI Malawi; SEN Senegal; RR relative risk; CI confidence intervals. (TIF 1732 kb) [file 12937_2018_382_MOESM1_ESM.tif]

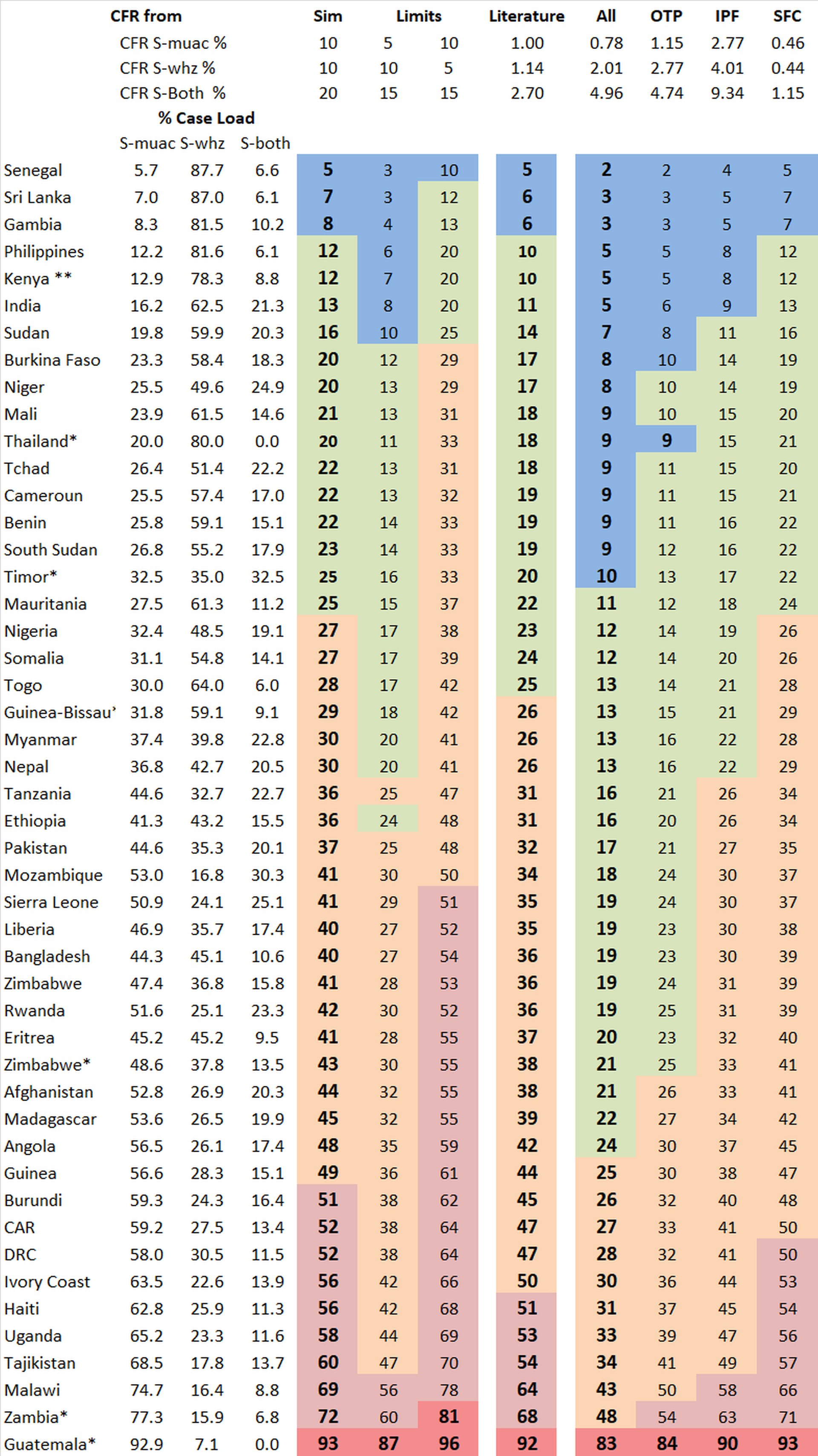

Supplement: Supplementary file 2 — Figure S2. Percentage of SAM-related-deaths of children that would be excluded from treatment by a WHZ-only program. Sim simulation data from Fig. 1, representing the likely extremes and probable ratio of case fatality rates (CFRs); All, IPF, OPT, SFC are the empirical case fatality rates of patients under different modes of treatment [16]; Literature mortality rates from Additional file S1, from reference [17]; Case Loads S-muac = MUAC < 115 mm with WHZ > = −3Z: S-whz = WHZ < −3Z with MUAC > 115 mm: S-both = MUAC < 115 mm and WHZ < −3Z; DRC Democratic Republic of the Congo; CAR Central African Republic. The case loads per country are from reference [12]. The colours represent the percent of total SAM-related-deaths occurring in cases that would be excluded from treatment in a MUAC-only program: Red 75–100%: Pink 50–75%: Orange 25–50%: green 10–25%: Blue 0–10%. * These countries case load comes from a small sample size. ** The case load from Kenya comes from the North of Kenya (similar to Sahel). (TIF 4541 kb) [file 12937_2018_382_MOESM2_ESM.tif]
